# Accelerated Theta Burst Transcranial Magnetic Stimulation for Refractory Depression in Autism Spectrum Disorder

**DOI:** 10.1007/s10803-024-06244-2

**Published:** 2024-05-15

**Authors:** Elizabeth Blank, Donald L. Gilbert, Steve W. Wu, Travis Larsh, Rana Elmaghraby, Rui Liu, Elizabeth Smith, Grace Westerkamp, Yanchen Liu, Paul S. Horn, Ethan Greenstein, John A. Sweeney, Craig A. Erickson, Ernest V. Pedapati

**Affiliations:** 1 Division of Child and Adolescent Psychiatry, Cincinnati Children’s Hospital Medical Center, Cincinnati, OH United States; 2 Division of Neurology, Cincinnati Children’s Hospital Medical Center, Cincinnati, OH United States; 3 Department of Psychiatry, University of Cincinnati College of Medicine, Cincinnati, OH United States; 4 Division of Behavioral Medicine and Child Psychology, Cincinnati Children’s Hospital Medical Center, Cincinnati, OH United States

**Keywords:** Autism, Depression, Fluid cognition, Transcranial Magnetic Stimulation, Brain Stimulation, Neuromodulation

## Abstract

**Purpose:**

Major depressive disorder (MDD) disproportionately affects those living with autism spectrum disorder (ASD) and is associated with significant impairment and treatment recidivism.

**Methods:**

We studied the use of accelerated theta burst stimulation (ATBS) for the treatment of refractory MDD in ASD (3 treatments daily x 10 days). This prospective open-label 12-week trial included 10 subjects with a mean age of 21.5 years, randomized to receive unilateral or bilateral stimulation of the dorsolateral prefrontal cortex.

**Results:**

One participant dropped out of the study due to intolerability. In both treatment arms, depressive symptoms, scored on the Hamilton Depression Rating Scale scores, diminished substantially. At 12 weeks post-treatment, full remission was sustained in 5 subjects and partial remission in 3 subjects. Treatment with ATBS, regardless of the site of stimulation, was associated with a significant, substantial, and sustained improvement in depressive symptomatology via the primary outcome measure, the Hamilton Depression Rating Scale. Additional secondary measures, including self-report depression scales, fluid cognition, and sleep quality, also showed significant improvement. No serious adverse events occurred during the study. Mild transient headaches were infrequently reported, which are expected side effects of ATBS.

**Conclusion:**

Overall, ATBS treatment was highly effective and well-tolerated in individuals with ASD and co-occurring MDD. The findings support the need for a larger, sham-controlled randomized controlled trial to further evaluate efficacy of ATBS in this population.

Autistic individuals are disproportionately affected by major depressive disorder (MDD), which contributes to functional disability, including educational and vocational impairment, social withdrawal, and suicide across the lifespan (Cassidy et al., 2014; Hollocks et al., 2019; Hudson et al., 2019; Matson & Nebel-Schwalm, 2007). Suicidal ideation in ASD is also elevated, with over 72% of adults with ASD scoring above the recommended cut-off for suicide risk on the Suicide Behaviors Questionnaire-Revised (SBQ-R) which was significantly correlated with reported non-suicidal self-injury, camouflaging, and number of unmet support needs (Cassidy et al., 2018). Though MDD is prevalent in ASD regardless of cognitive ability, autistic individuals without intellectual disability disorder (IDD) are more likely to receive the diagnosis, likely due to clearer communication of internal states and more canonical presentations (Pezzimenti et al., 2019).

A major challenge in treating MDD among those with ASD is the high rates of treatment recidivism and relapse (Hirvikoski et al., 2016). It is estimated that treatment-resistant MDD in ASD individuals likely exceeds that of the general population (> 30%) based on prevalence rates and polypharmacy (Feroe et al., 2021; Rosenberg et al., 2010; Zheng et al., 2021). This is consistent with observations that standard-of-care medications for mood disorders can be unpredictable in ASD and may at times even be counterproductive in ameliorating symptoms (McCracken et al., 2021; Williams et al., 2013). Despite the urgent need for support for this population, little research exists targeting novel interventions for depression and suicidality in ASD.

Repetitive transcranial magnetic stimulation (rTMS) is an evidence-based intervention for MDD in typically developing populations (Razza et al., 2018). rTMS involves brief, high-intensity electrical currents passing through a coil placed near the scalp. This induces a rapidly changing magnetic field that induces an electrical current in local brain parenchyma, leading to both local inhibitory or excitatory neuronal changes as well as changes in connected brain regions (Terao & Ugawa, 2002). Though meta-analysis of rTMS randomized sham-controlled trials demonstrate efficacy in treatment of MDD severity and remission rates, several factors limit the feasibility of the conventional form of treatment in autistic individuals (Sehatzadeh et al., 2019). First, although patients remain awake during the procedure and require no aftercare, traditional rTMS stimulation is delivered above resting motor threshold (RMT) and can lead to headaches, scalp pain, muscle twitching, and eye discomfort. In ASD cohorts, where sensory hypersensitivity is more common, the prevalence of AEs associated with TMS is estimated to be at 25% (Huashuang et al., 2022). Moreover, a conventional rTMS treatment course typically involves daily 45-minute treatment sessions spanning four to six weeks, requiring a significant investment of time and logistical coordination.

Recent advances in rTMS protocols, namely theta burst stimulation (TBS) may help improve tolerability in which sensory hypersensitivity or duration of treatment may be a limiting factor (Elmaghraby et al., 2022; Hong et al., 2015). Since TBS protocols use a higher frequency pulse (> 30 Hz), they only involve several minutes of stimulation and can be performed at or below RMT, minimizing overall sensation (Huang et al., 2005b). Additionally, multiple treatments of TBS or accelerated TBS (ATBS) can be performed in a single day which can dramatically shorten the overall duration of treatment to one to ten days (Duprat et al., 2016; Fitzgerald et al., 2020; Weissman et al., 2018a). So far, TBS protocols (with or without acceleration) are comparable in safety and efficacy to conventional rTMS, but they offer advantages in tolerability, treatment capacity, and cost-effectiveness (Blumberger et al., 2018; Cai et al., 2023). The US Food and Drug Administration (FDA, 2011) cleared the use of TBS in 2018 and ATBS in 2022 as an alternative to conventional rTMS for MDD (Neuteboom et al., 2023).

No RCTs evaluating the efficacy of any form of TMS treatment of MDD in individuals with ASD are available. However, a recent open-label trial of conventional rTMS for MDD in adults with ASD (*n* = 10) found that 70% of participants responded to treatment and 40% reached remission (Gwynette et al., 2020). Two participants withdrew due to intolerability. Participants with sensitivity to stimulation were started on a lower stimulation intensity and gradually titrated to the full dose or used a < 1 mm foam barrier at the stimulation site. While these results are promising, we hypothesized that the abbreviated course and reduced stimulation intensity of ATBS may be better suited for individuals with ASD.

We conducted a prospective open-label accelerated TBS on treatment-refractory MDD in transition-aged youth with ASD (ages 12–26 years). The Hamilton Depression Rating Scale (HRDS-17) was used as the primary outcome, and we assessed changes from baseline at 1-, 4-, and 12-weeks post-treatment. To investigate stimulation parameters, we randomized participants either unilateral (UL) left dorsolateral prefrontal cortex (DLPFC) stimulation or bilateral stimulation (BL) DLPFC based on recent literature suggesting potential advantages of bilateral stimulation (Blumberger et al., 2012; Chistyakov et al., 2015). Our hypothesis was that bilateral stimulation would enhance the treatment efficacy but may also negatively affect tolerability. Additionally, we administered NIH Cognitive Toolbox measures at each timepoint hypothesizing that changes in scores may reflect prefrontal cortex engagement and predict MDD treatment response (Crane et al., 2017).

## Methods

### Ethics Statement

This study was approved by the institutional review board at Cincinnati Children’s Hospital Medical Center (CCHMC) and registered with ClinicalTrials.gov (NCT01609374). Recruitment took place between November 2021 and November 2022 through clinician referrals, community flyers, emails, and clinics at a tertiary academic pediatric hospital. All participants provided written informed consent or assent for all study procedures.

Diagnosis of MDD and co-occurring conditions was determined by the Mini-International Neuropsychiatric Interview for Children and Adolescents (MINI-KID) for participants under 18 years of age (Sheehan et al., 1998) and the Structured Clinical Interview for DSM-5 Disorders (SCID-5) for those 18 and older (Spitzer et al., 1992). Treatment-resistant MDD was determined using the Antidepressant Treatment History Form (Sackeim et al., 2019). Diagnostic assessments were conducted by qualified experienced raters, including licensed clinical psychologists (ADOS-2, MINI-KID) or board-certified child and adolescent psychiatrists (MINI-KID, ATHF).

### Participants

Inclusion criteria for participants included: (1) age 12–26 years, (2) diagnosed with ASD (and confirmed by Autism Diagnostic Observation Schedule, 2nd Edition (ADOS-2) (Lord et al., 2012), (3) currently meeting Diagnostic and Statistical Manual of Mental Disorders, Fifth Edition (DSM-5) criteria for a unipolar major depressive disorder or persistent depressive disorder, 3) exhibiting treatment resistance to at least one evidence-based antidepressant medication, (4) Global Assessment of Function (GAF) score ≤ 60, (5) 17-item Hamilton Depression Rating Scale (HDRS-17) or Beck Depression Inventory II (BDI-II) score in the clinically depressed range (≥ 20) that was sustained over the two-week lead-in period.

Exclusion criteria included any of the following: (1) significant psychiatric or neurological disease unrelated to ASD or MDD within the last six months, (2) use of investigational drugs, (3) any contraindications to TMS (Rossi et al., 2021) (4) Intelligence Quotient < 80 per the Wechsler Abbreviated Scale of Intelligence, 2nd Edition (Wechsler, 1999), (5) active pregnancy (confirmed by urine test), (6) active suicidality, (7) history of epilepsy or use of antiepileptic drugs, (8) prior rTMS treatment, (9) changes in psychiatric medicines two weeks before TMS treatment, 11) substance use or substance dependence disorder (confirmed by urine toxicology).

### Study Design

**Fig. 1:**
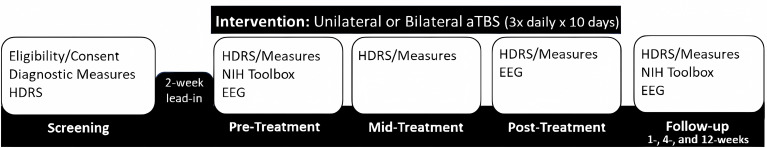
Accelerated theta burst stimulation (aTBS) randomized control study design. Participants were assessed at seven timepoints up to 12-weeks following treatment

This open-label prospective clinical trial involved 30 TBS sessions over a period of ten days (Fig. 1). Following the screening visit, eligible participants were randomly assigned to receive either standard, unilateral intermittent TBS (iTBS) to the left dorsolateral prefrontal cortex (DLPFC) (FDA, 2011) or bilateral stimulation with iTBS to the left DLPFC and continuous TBS (cTBS) to the right DLPFC. Following randomization, participants had to maintain eligibility for a two-week lead-in period prior to the first treatment session. Additional assessments were conducted at days 5 and 10 of the intervention and 1-, 4-, and 12-weeks post-treatment.

### Intervention

A Magstim Horizon Performance stimulator (Magstim, Whitland, UK) with a 70mmm figure-eight EZ cool coil was used for all treatment sessions. Coil placement was determined using the BEAM-F3 method (Beam et al., 2009). A separate figure-eight coil was used to establish RMT. RMT was defined as the lowest TMS intensity needed to produce a contralateral thumb twitch in at least three of six trials (Horvath et al., 2010). For participants in the BL group, RMT was determined for each hemisphere. All iTBS and cTBS sessions consisted of triplet 50 Hz pulses repeated in 5 Hz bursts for a total of 600 pulses per session at 90% of RMT (Huang et al., 2005a). During iTBS 20 trains were applied in 2-second bursts with 8-second pauses, while cTBS involved a continuous pulse train for a total duration of 53 s. TBS was delivered in three sessions daily over ten days, with 50 min intervals between sessions (Cai et al., 2023). To account for participants with sensory hypersensitivity we titrated up to target (90%) stimulation intensity over the first two treatment days, starting at 50% RMT and increasing by 10–20% each session, depending on each subject’s tolerance.

### Outcome Measures

The primary outcome measure was change in scores on the HDRS-17 (Hamilton, 1986). Secondary depression measures (for validation) included BDI-II (Osman et al., 2004) and Quick Inventory of Depressive Symptomatology (QIDS) (Rush et al., 2003). Suicidal behavior was assessed by physician-administered Columbia-Suicide Severity Rating Scale (C-SSRS) (Posner et al., 2008) at screening and self-report Suicide Behavior Questionnaire (SBQ) (Osman et al., 2001) at screening, intervention days 5 and 10, and all follow-up visits. Changes in anxiety symptoms were assessed using the Generalized Anxiety Disorder-7 item (GAD-7) (Spitzer et al., 2006). Changes in sleep were measured using the Pittsburgh Sleep Quality Inventory (PSQI) (Buysse et al., 1989), and social functioning was measured using the Social Responsiveness Scale (SRS) (Bruni, 2014). Neurocognitive function was assessed using the NIH Cognitive Toolbox (processing speed, working memory, language, and executive function i.e., inhibitory control, set shifting) (Weintraub et al., 2013) and neuromuscular function using the Grip Strength Test from the NIH Toolbox Motor Battery (Reuben et al., 2013).

### Safety Outcomes

To assess adverse events (AEs) related to TMS, a 16-point systematic review of systems was conducted at the beginning and end of each treatment day.

### Statistical Analysis

Given the exploratory nature of this pilot study, we present data at the individual level and used streamlined statistical modeling to discern overarching trends and effects. No outliers were detected. For each outcome measure, we provide a three-panel figure depicting:

1. Main Effect Plot: This plot displays the mean values of the measure across different time points.
2. Group Interaction Plot: This plot illustrates the interaction effect between time and stimulation site. The mean values of the measure are plotted, stratified by treatment.
3. Subject-level Plot: This plot provides insights into individual variability by plotting each subject’s mean value for the measure across time.

We conducted a linear mixed-effects analysis using the *lmerTest* package in R 4.3 to identify any main effects of time or treatment arm, as well as their potential interaction effect on each outcome measure. To account for the repeated measures design, we incorporated random intercepts for subjects.

Mathematically, the model can be represented as:

$${Y}_{ijk}=\mu +{\alpha }_{i}+{\beta }_{j}+(\alpha \beta {)}_{ij}+{\gamma }_{k}+{\epsilon _{ijk}}$$

Where:

- ${Y}_{ijk}$ is the dependent variable (e.g., a specific measure for a given subject at a particular time in a certain group).
- $\mu$ is the overall mean.
- ${\alpha }_{i}$ represents the effect of the ith level of factor A (Time).
- ${\beta }_{j}$ denotes the effect of the jth level of factor B (Group).
- $(\alpha \beta {)}_{ij}$ stands for the interaction effect between the ith level of factor A and the jth level of factor B.
- ${\gamma }_{k}$ is the random effect of the kth subject (or individual).
- ${\epsilon}_{ijk}$ is the random error associated with the kth observation under the ith level of factor A and jth level of factor B.

Following model estimation, we extracted the ANOVA table to ascertain if a main or interaction effect was present. Depending on the effect, post-hoc tests were carried out to assess pairwise differences between baseline and post-treatment time points, while also using false discovery rate (FDR) to adjust for multiple comparisons. An adjusted *p* value less than or equal to 0.05 was considered statistically significant.

## Results

**Fig. 2:**
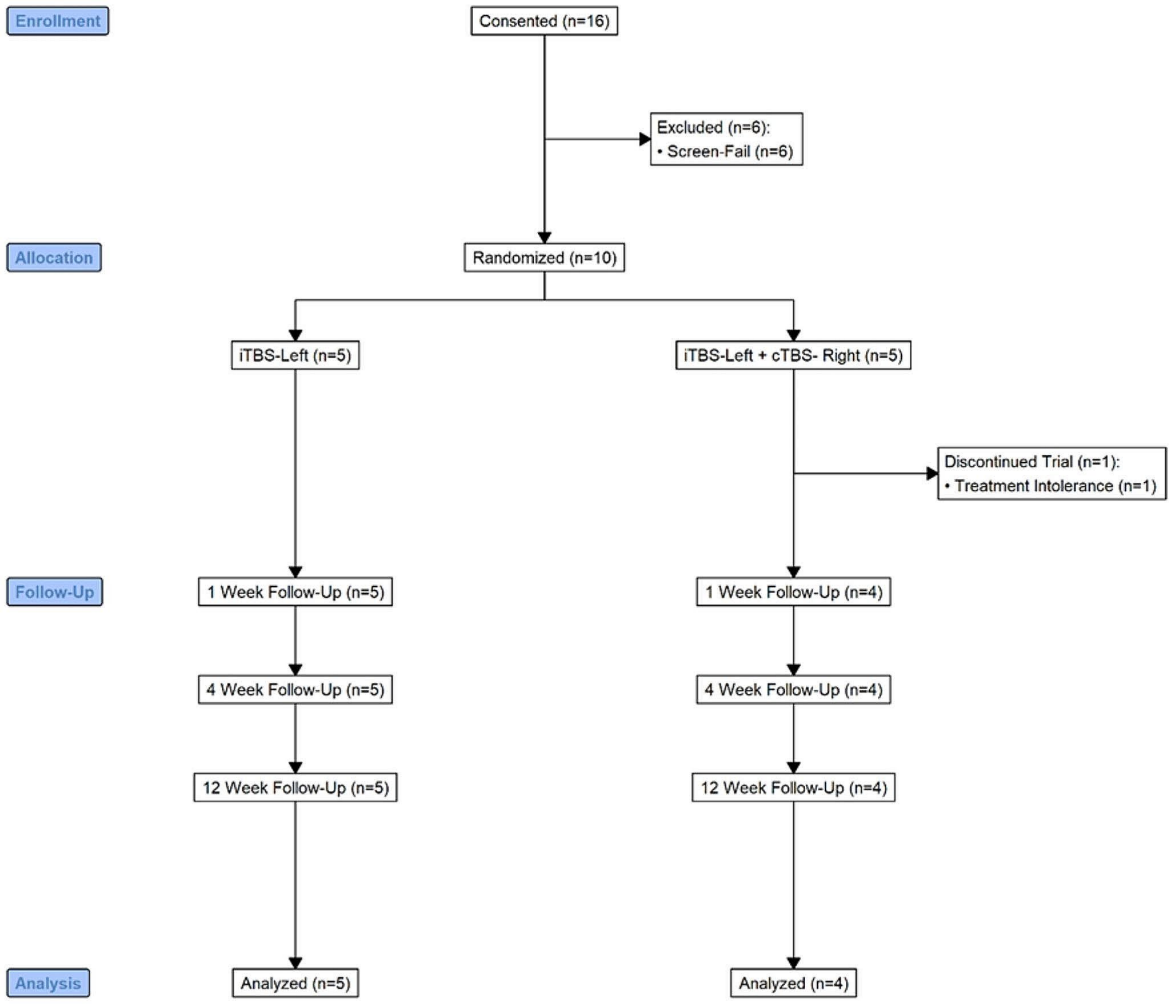
CONSORT Flow Diagram

Ten participants (2 females; min age = 17, max age = 26.2, median age = 22) with ASD and treatment refractory MDD (mean failed antidepressant trials = 3.44 ± 1.7) were randomized to either UL or BL ATBS treatment. Demographics and baseline clinical measures (including MDD severity) were similar between treatment arms (Table 1). One subject disclosed additional history during the trial that supports a diagnosis of borderline personality disorder. While the subject was included in all the main analysis models, they were excluded from the exploratory correlation analysis. Consolidated Standards of Reporting Trials (CONSORT) flow diagram showing the selection of participants from initial screening to final analysis (Fig. 2).

**Table 1:** Baseline demographic and clinical characteristics of the study participants

Measure	Combined, n = 9	Unilateral, n = 5	Bilateral, n = 4	*p*-value
Age	21.5 ± 3.2	21.7 ± 3.9	21.2 ± 2.6	> .99
Sex				> .99
Male	7 (78%)	4 (80%)	3 (75%)	
Female	2 (22%)	1 (20%)	1 (25%)	
Race				.29
White	6 (67%)	2 (40%)	4 (100%)	
Black or African American	1 (11%)	1 (20%)	(0%)	
Asian	2 (22%)	2 (40%)	(0%)	
American Indian, Alaskan Native, Native Hawaiian, or other Pacific Islander	0 (0%)	(0%)	(0%)	
Ethnicity				.44
Hispanic or Latino	1 (11%)	(0%)	1 (25%)	
Not Hispanic or Latino	8 (89%)	5 (100%)	3 (75%)	
Full-Scale IQ	113.4 ± 12.5	113.4 ± 14.7	113.5 ± 11.4	> .99
Primary diagnosis				> .99
Dysthymia	2 (22%)	1 (20%)	1 (25%)	
MDD	7 (78%)	4 (80%)	3 (75%)	
# of failed antidepressant trials	3.4 ± 1.7	3.8 ± 2.2	3.0 ± 1.2	.46
SRS-II total	103.6 ± 25.8	103.8 ± 33.7	103.2 ± 16.3	> .99
Vineland-3 Composite Score	76.1 ± 8.1	76.8 ± 10.0	75.0 ± 5.0	> .99
Communication domain	80.3 ± 4.5	81.2 ± 3.7	78.7 ± 6.0	> .99
Receptive domain	12.0 ± 1.6	12.6 ± 1.1	11.0 ± 2.0	> .99
Daily Living Skills domain	83.8 ± 10.7	85.2 ± 12.0	81.3 ± 9.9	> .99
Socialization domain	69.1 ± 19.4	68.6 ± 25.4	70.0 ± 5.0	> .99
BRIEF, Global Executive Composite	143.3 ± 17.0	140.0 ± 20.8	147.5 ± 12.3	> .99
GAF	51.1 ± 6.1	52.6 ± 4.2	49.2 ± 8.3	.71
HDRS-17 total score	20.2 ± 3.4	19.6 ± 2.5	21.0 ± 4.5	.44
BDI-II total score	25.3 ± 14.3	23.4 ± 18.4	27.8 ± 8.9	> .99
QIDS total score	19.1 ± 4.6	19.2 ± 5.0	19.0 ± 4.8	.81
CGI-Severity	4.9 ± 0.3	4.8 ± .4	5.0 ± .0	> .99
EDI-Reactivity raw total	8.8 ± 5.6	8.8 ± 7.3	8.8 ± 3.7	.44
EDI-Dysphoria raw total	13.4 ± 7.8	14.0 ± 6.6	12.8 ± 10.2	.68
RRS total score	24.9 ± 5.4	26.0 ± 6.3	23.5 ± 4.5	> .99
GAD total score	12.1 ± 4.7	13.0 ± 4.7	11.0 ± 5.1	.68
PSQI global score	11.3 ± 4.3	11.6 ± 5.0	11.0 ± 3.9	.44
SBQ-R total score	9.9 ± 4.5	10.0 ± 4.0	9.8 ± 5.7	.44
Fluid Cognition Composite raw score	105.8 ± 10.4	103.0 ± 12.4	109.2 ± 7.3	> .99
Grip Strength (lbs. force)	66.6 ± 12.7	72.0 ± 6.8	59.7 ± 15.9	> .99

Data are presented as either mean ± SD or n (%) for Combined (*n* = 9), Unilateral (*n* = 5), and Bilateral (*n* = 4) groups. Measures include age, sex, race, ethnicity, IQ, primary diagnosis, and various clinical measures

### Clinical and Behavioral Outcomes

Summary results of the effects from each LME model are displayed in Table 2. For models that showed a significant main or interaction effect, post-hoc testing results are presented in Table 3.

**Table 2:** Results of mixed-effects linear models assessing the effects of time, group, and their interaction on clinical measures

Measure	Effect	Num DF	Den DF	MS	SS	F	*p*-value	Sig
HRDS-17 Total Score	Time	3	21	328.94	986.82	28.49	< 0.001	***
	Group	1	7	1.67	1.67	0.14	0.715	
	Time*Group	3	21	9.68	29.04	0.84	0.488	
BDI-II Total	Time	3	20	202.79	608.38	9.36	< 0.001	***
	Group	1	7	0.74	0.74	0.03	0.858	
	Time*Group	3	20	17.85	53.54	0.82	0.496	
QIDS Total	Time	3	21	155.50	466.50	7.72	0.001	**
	Group	1	7	1.05	1.05	0.05	0.826	
	Time*Group	3	21	8.83	26.50	0.44	0.728	
GAD-7 Total	Time	3	21	35.15	105.45	3.71	0.028	*
	Group	1	7	0.67	0.67	0.07	0.799	
	Time*Group	3	21	12.04	36.12	1.27	0.310	
PSQI Score	Time	3	20	32.66	97.99	3.66	0.030	*
	Group	1	7	1.31	1.31	0.15	0.713	
	Time*Group	3	20	9.06	27.18	1.02	0.406	
SRS Total	Time	3	21	68.62	205.86	0.80	0.507	
	Group	1	7	1.31	1.31	0.02	0.905	
	Time*Group	3	21	74.10	222.30	0.87	0.474	
Fluid Cognition Raw Score	Time	3	21	312.79	938.37	10.00	< 0.001	***
	Group	1	7	69.56	69.56	2.22	0.180	
	Time*Group	3	21	21.23	63.70	0.68	0.575	
Grip Strength Measurement (lbs. force)	Time	3	21	93.28	279.84	1.19	0.339	
	Group	1	7	444.27	444.27	5.65	0.049	*
	Time*Group	3	21	67.48	202.43	0.86	0.478	

The table includes the degrees of freedom (Num DF for effects, Den DF for error), Mean Squares (MS), Sum of Squares (SS), F-statistic values (F), Effect Size (EF) as Cohen’s F, and *p*-values (p) for each effect. The ‘sig’ column indicates significance levels (* *p* < 0.05, ** *p* < 0.01, *** *p* < 0.001). Each row represents a different measure or interaction. The Cohen’s F effect size (EF) correspond to small (0.10), medium (0.25), and large (0.40). The measures assessed include Hamilton Rating Scale for Depression (HRDS-17 Total Score), Beck Depression Inventory-II (BDI-II Total), Quick Inventory of Depressive Symptomatology (QIDS Total), Generalized Anxiety Disorder 7-item (GAD-7 Total), Pittsburgh Sleep Quality Index (PSQI Score), Social Responsiveness Scale (SRS Total), NIH Toolbox Fluid Cognition, and Grip Strength Measurement

**Table 3:** Results of post hoc tests, conducted to investigate significant model effects

Measure	vs. Baseline	DF	Estimate	t	*p*-value	Sig
HRDS-17 Total Score	Week 1	21.00	11.42	7.09	< 0.001	***
	Week 4	21.00	12.58	7.80	< 0.001	***
	Week 12	21.00	12.38	7.68	< 0.001	***
BDI-II Total	Week 1	20.00	8.20	3.71	0.007	**
	Week 4	20.00	9.52	4.31	0.002	**
	Week 12	20.02	10.65	4.66	< 0.001	***
QIDS Total	Week 1	21.00	7.65	3.59	0.009	**
	Week 4	21.00	8.93	4.19	0.002	**
	Week 12	21.00	8.33	3.91	0.004	**
GAD-7 Total	Week 1	21.00	4.03	2.76	0.056	
	Week 4	21.00	3.45	2.36	0.123	
	Week 12	21.00	4.28	2.93	0.039	*
PSQI Score	Week 1	20.00	3.35	2.37	0.124	
	Week 4	20.00	4.38	3.09	0.028	*
	Week 12	20.17	3.51	2.34	0.130	
Fluid Cognition Raw Score	Week 1	21.00	-7.37	-2.78	0.053	
	Week 4	21.00	-8.05	-3.03	0.031	*
	Week 12	21.00	-14.50	-5.47	< 0.001	***

These tests examine differences between Baseline and subsequent time points (Week 1, Week 4, and Week 12) across various measures. All post-hoc tests underwent a 5% FDR *p*-value adjustment. The table provides information on degrees of freedom (DF), estimated differences from the baseline (Estimate), t-statistic values (t), adjusted *p*-values (Adj. p.) for controlling the family-wise error rate, and the significance (Sig) of the comparisons (* *p* < 0.05, ** *p* < 0.01, *** *p* < 0.001). The measured variables include the Hamilton Rating Scale for Depression (HRDS-17 Total Score), Beck Depression Inventory-II (BDI-II Total), Quick Inventory of Depressive Symptomatology (QIDS Total), Generalized Anxiety Disorder 7-item (GAD-7 Total), Pittsburgh Sleep Quality Index (PSQI Score), and NIH Toolbox Fluid Cognition

### Depression: Primary and Secondary Outcomes

**Fig. 3:**
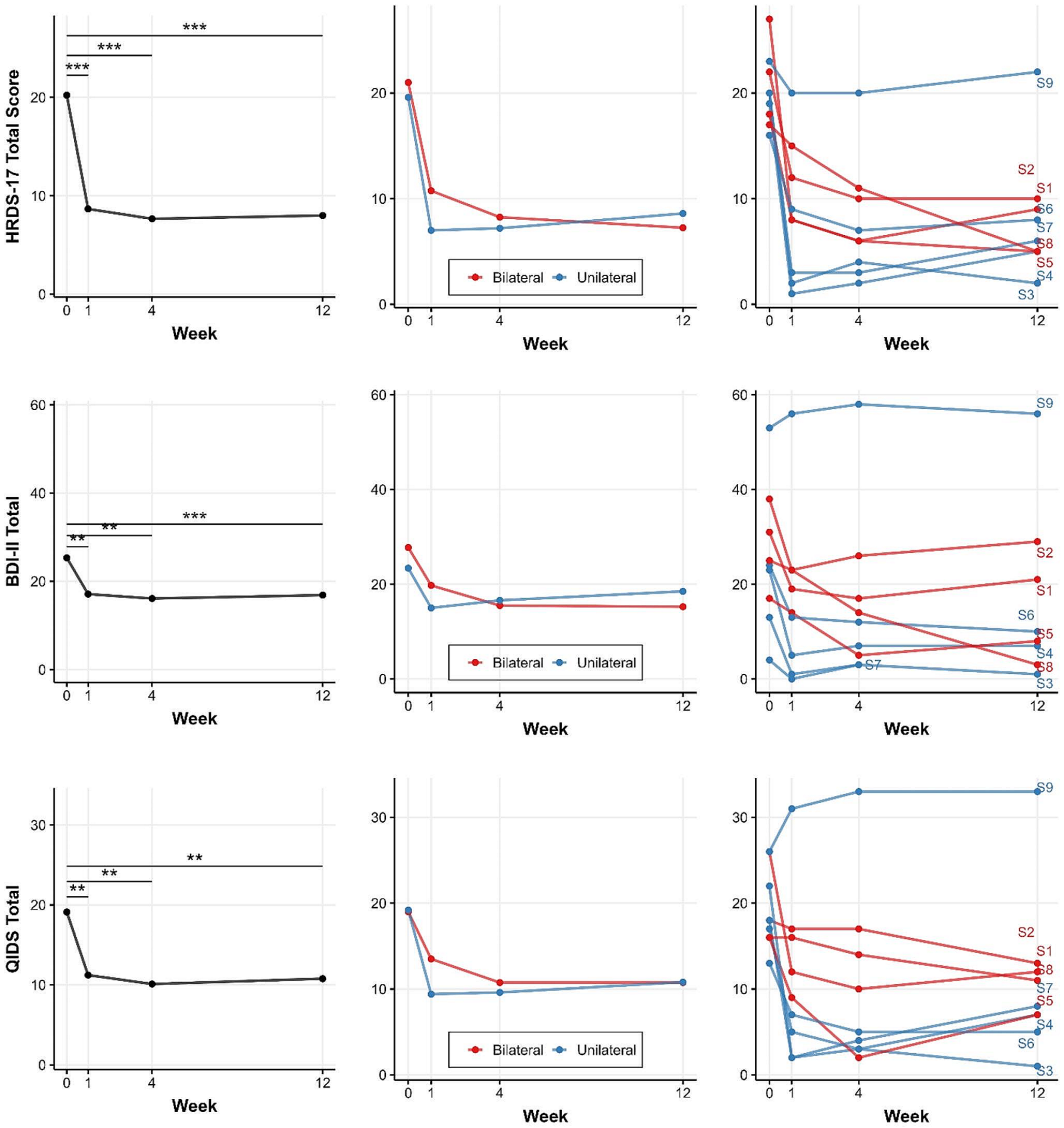
Trajectories on depression scales (HDRS-17 Total Score, BDI-II Total, QIDS Total) over 12 weeks following ATBS treatment. Timepoint 0: baseline; Timepoints 1, 4, and 12: post-treatment follow-ups, numbered by week. Left: group means with significance; center: Bilateral vs. Unilateral group averages; right: individual progressions (S1-S9). Significance levels: * *p* < 0.05, ** *p* < 0.01, *** *p* < 0.001

The main effect of time was significant for the primary outcome of interest, HRDS-17, F(3, 21) = 28.49, *p* < 0.001, EF = 1.976, indicating a large Cohen’s treatment effect size (EF). For HRDS-17 Total Score, significant differences from baseline were observed at all three time points: Week 1, t(21) = 7.09, *p* < 0.001, estimated change = -11.43; Week 4, t(21) = 7.80, *p* < 0.001, estimated change = -12.58; Week 12, t(21) = 7.68, *p* < 0.001, estimated change = -12.38. By week 4, 7 out of 9 subjects showed a significant treatment response (≥ 50% reduction from baseline HDRS-17 score). Treatment effects were largely sustained through the 12-week follow-up period (Fig. 3); at this timepoint, five subjects met the criteria for full remission of depression and three achieved partial remission (American Psychiatric Association & Association, 2013).

Only the main effect of time was significant for BDI-II Total, F(3, 20) = 9.36, *p* < 0.001, EF = 1.181, also indicating a large treatment effect. Similarly, for QIDS Total, only the main effect of time was significant, F(3, 21) = 7.72, *p* = 0.001, EF = 1.037. Post-hoc tests (Table 3) demonstrated that BDI and QIDS saw significant improvements from baseline at Weeks 1, 4, and 12 (*p* < ﻿0.01 for each).

### Anxiety

**Fig. 4:**
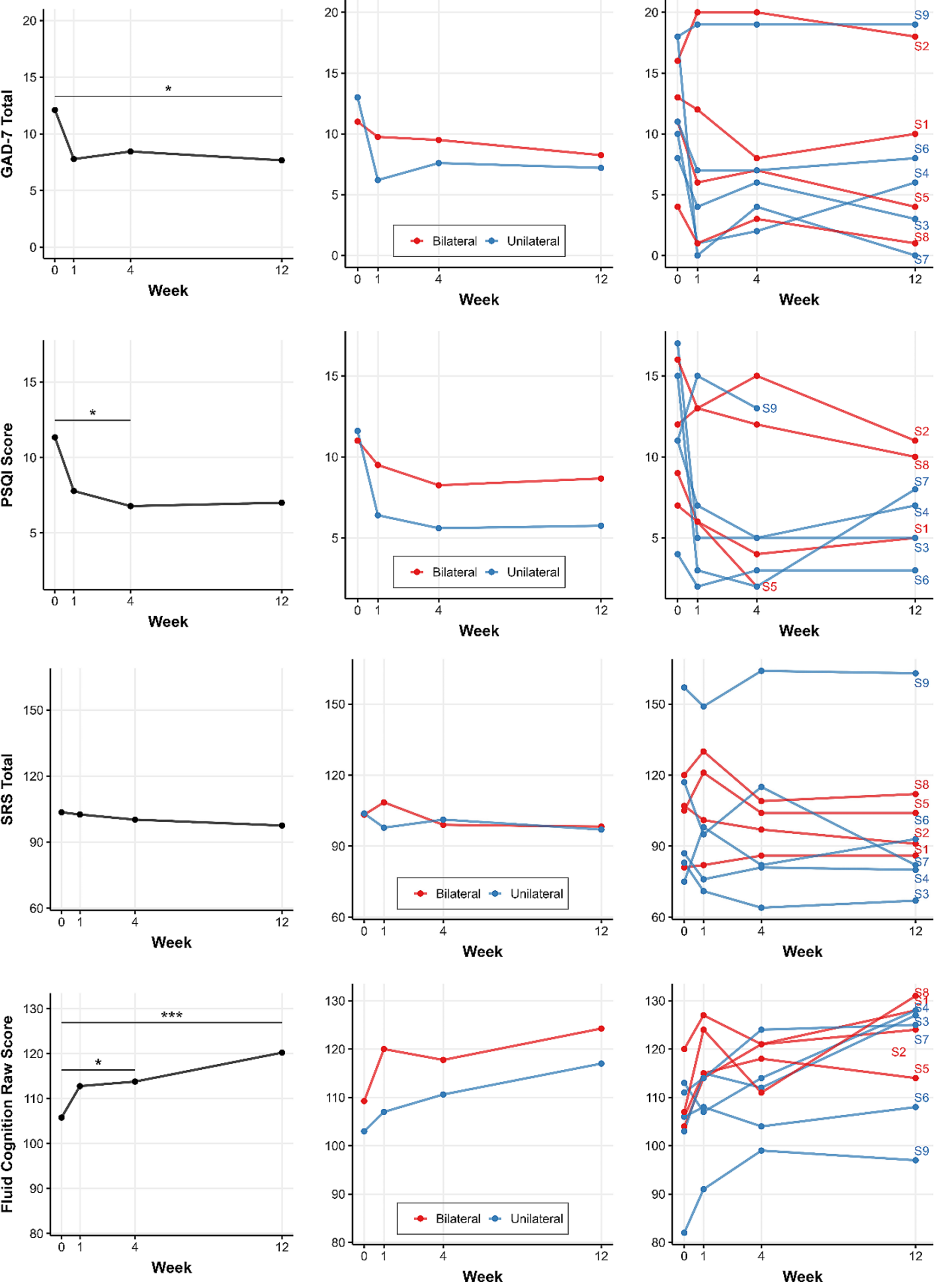
Longitudinal score changes for various clinical measures (GAD-7 Total, PSQI Score, SRS Total, Fluid Cognition Raw Score) over 12 weeks following ATBS treatment. Timepoint 0: baseline; Timepoints 1, 4, and 12: post-treatment follow-ups, numbered by week. Left: group means with significance; center: Bilateral vs. Unilateral group averages; right: individual progressions (S1-S9). Significance levels: * *p* < 0.05, ** *p* < 0.01, *** *p* < 0.001

For GAD-7 Total, the main effect of time was significant, F(3, 21) = 3.71, *p* = 0.028, EF = 0.721 (Fig. 4). Post-hoc tests showed a significant difference from baseline observed at Week 12: t(21) = 2.93, *p* = 0.039, estimated change = 4.27, but not for Week 1 or 4.

### Sleep

For PSQI Score, the main effect of time was significant, F(3, 19) = 4.27, *p* = 0.018, EF = 0.799 and demonstrated a large treatment effect (Fig. 4). Post-hoc tests demonstrated a significant improvement in sleep ratings at Week 4: t(19) = 3.19, *p* = 0.023, estimated change = 4.38 and trending improvement at Week 12.

### Social

For SRS Total, neither the main effect of time, F(3, 21) = 0.80, *p* = 0.51, EF = 0.337, nor the interaction between time and group, F(3, 21) = 0.87, *p* = 0.47, EF = 0.350, were significant (Fig. 4). Neurocognitive.

For Fluid Cognition, only the main effect of time was significant, F(3, 21) = 10.00, *p* < 0.001, EF = 1.174, indicating a large effect size (Fig. 4). Post-hoc testing found a trending improvement in Fluid Cognition from baseline to Week 1 and significant improvement from baseline were observed at Week 4, t(21) = -3.03, *p* = 0.031, estimated change = -8.05, and Week 12, t(21) = -5.47, *p* < 0.001, estimated change = -14.50.

### Neuromuscular

For Grip Strength, neither the main effect of time, F(3, 21) = 1.19, *p* = 0.34, EF = 0.394, nor the interaction between time and group, F(3, 21) = 0.86, *p* = 0.48, EF = 0.335, were significant, but the main effect of group was trending, F(1, 7) = 5.65, *p* = 0.05, EF = 0.497.

### Exploratory Biomarker

**Fig. 5:**
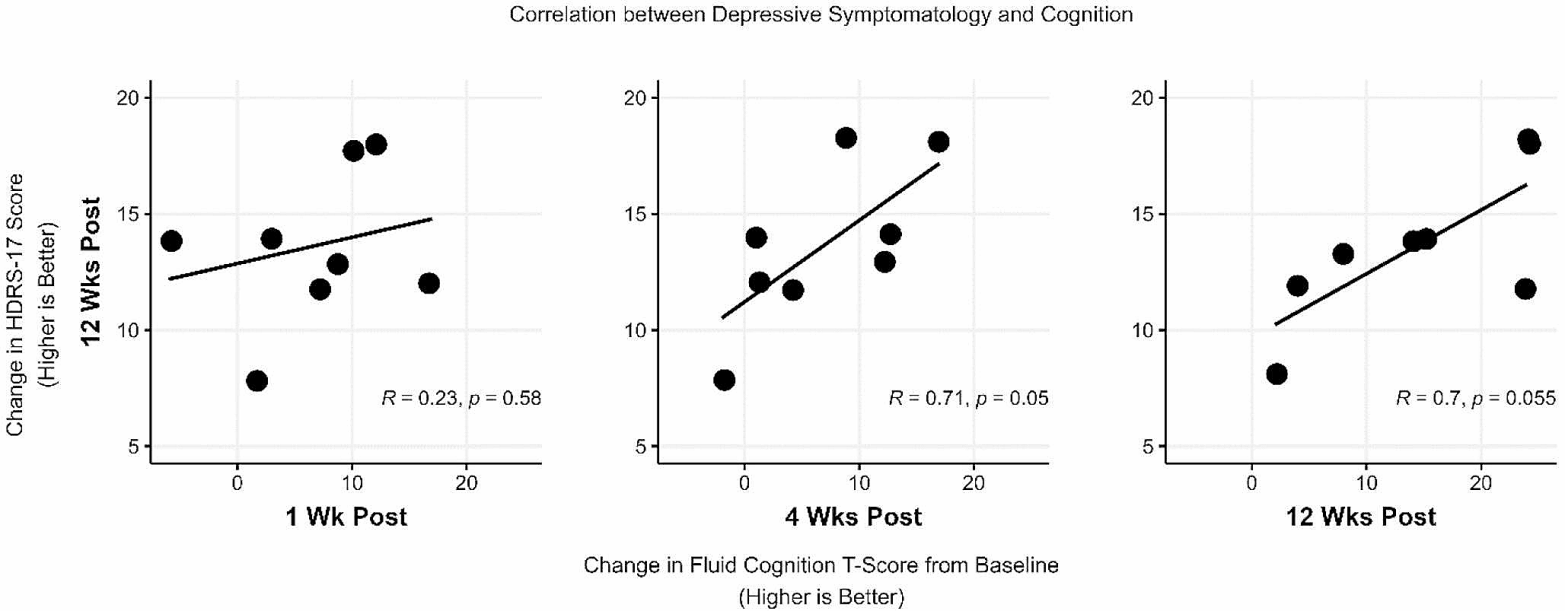
Correlation between 12-week changes in HDRS-17 scores and changes in Fluid Cognition T-Scores at 1, 4, and 12 weeks post-treatment. Lines of best fit with Spearman coefficients and *p*-values indicate the strength and significance of the correlations

An exploratory analysis was conducted to investigate the relationship between changes in Fluid Cognition scores (NIH Toolbox) and changes in depressive symptomatology (measured by HRDS-17) at Week 12. The results indicated a significant correlation between improvement in Fluid Cognition scores at week 4 and reduction in depression symptoms at 12 (Fig. 5).

### Safety Outcomes

The ATBS intervention was completed by nine subjects (UL = 5; BL = 4). However, one participant in the bilateral group was unable to tolerate the stimulation and withdrew from treatment. Five subjects (*n* = 2 UL; *n* = 3 BL) required titration to reach target stimulation intensity. Overall, adverse events were mild and self-limiting. Over the 90 treatment days (10 days per subject), a total of 6 headaches were reported, resulting in a 7% incidence rate. All headaches were associated with bilateral treatment and were described by participants as mild in severity and spontaneously resolved.

## Discussion

This open-label trial demonstrates the efficacy and safety of ATBS in transition-aged autistic youth with treatment-refractory MDD. Treatment-resistant MDD in ASD is a challenging condition to manage, often necessitating polypharmacy or therapeutic approaches associated with higher side effects. Though the efficacy of rTMS for MDD is well established, adapting the intervention at scale for ASD individuals poses unique challenges. The primary purpose of this study was to identify optimal design parameters and outcome measures to advance into a larger pivotal RCT. We observed a robust and sustained treatment in most participants following ATBS regardless of stimulation site. The observed statistically significant improvement across various MDD scales immediately post-treatment suggests ATBS may elicit rapid antidepressant effects, especially in comparison to antidepressant medications or psychotherapy and especially notable in a treatment-refractory sample.

It is important to consider the limitations of our study design, non-controlled studies have shown large effects in depression trials (Wager & Atlas, 2015; Walsh et al., 2002). Nevertheless, recent research on the placebo effect has suggested that it may activate similar areas of the brain as actual antidepressant treatment, and strong placebo effects may indicate regression to the mean. To mitigate these potential confounding factors and identify potential mechanisms, we collected high-resolution electroencephalography to assess changes in brain activity (in preparation) and the inclusion of a lead-in period to account for spontaneous remission of depression symptoms. There is considerable existing evidence that TMS treatment in depression is superior to sham stimulation in typically developing cohorts, albeit with recent meta-analyses lowering the effect size in some populations. (Brini et al., 2023). As the current FDA-cleared rTMS protocol does not exclude individuals with ASD, it remains an evidence-based treatment for MDD when typical therapies are ineffective(Zemplenyi et al., 2022). ATBS may be particularly well suited in cases where there is a high risk of adverse outcomes associated with prolonged depressive episodes, intolerability to typical rTMS, or when the next proximal step is electroconvulsive therapy.

We observed no significant difference in efficacy between Unilateral (UL) and Bilateral (BL) stimulation. However, BL stimulation was associated with a higher incidence of side effects and involved a greater number of procedures. The question of relative effectiveness of BL vs. unilateral rTMS in MDD treatment remains unclear; however, empirical data suggests that stimulation parameters, patient population, and tolerability should be considered (Blumberger et al., 2016; Fitzgerald et al., 2012, 2013; Trevizol et al., 2019; Weissman et al., 2018b). Taking into account the practical considerations which we observed and the present lack of evidence-based treatments, future RCTs may be well-served to focus on UL stimulation in ASD populations.

We administered serial computerized neurocognitive testing, hypothesizing that changes in performance on these tests could serve as biomarkers of prefrontal target engagement and predict later treatment effects (de Boer et al., 2021). Following ATBS treatment, we observed, in the majority of participants, a marked improvement in fluid cognition scores (NIH Toolbox) which was predictive of treatment response on the HRDS-17 at 12 weeks. The neurocognitive tests underlying these findings, such as the Flanker task and card sorting tasks, have been consistently linked to the efficiency of frontal-parietal cortical networks (Kim et al., 2017). However, in our sample, these findings are potentially confounded by concurrent improvements in MDD and associated pseudodementia (Kim et al., 2019).

Even if improvements of fluid cognition are unrelated to ATBS and instead secondary to the amelioration of MDD, assessing fluid cognition in ASD MDD may still serve as an indicator of the duration or response of treatment. Intriguingly, several studies have reported that, in comparison to typically developed individuals, those with ASD exhibit increased activity in temporal and occipital networks, but decreased activity in frontal-parietal networks during fluid reasoning tasks (Simard et al., 2015; Soulières et al., 2009). This suggests that independent studies of accelerated theta-burst stimulation for improving fluid cognition by engaging potentially underactive frontal-parietal networks in ASD may be worth exploring.

### Limitations

Several limitations to our study must be considered in context with the strong treatment effects. First and foremost, the potential of expectancy effects, widely observed in rTMS studies, particularly with younger subjects (Oberman et al., 2021; Xu et al., 2023), is important to consider. However, the observed changes in fluid cognition provide an objective marker of treatment response, thus reducing the likelihood of expectancy effects significantly influencing our results. Second, sample size and the exclusion of autistic individuals with IDD reduces the power, generalizability of these results, and our ability to identify subgroups of patients who may benefit most from ATBS compared to other forms of treatment. For example, one participant disclosed during treatment additional history and symptoms consistent with co-occurring personality disorder and did not demonstrate any treatment effects. This result is consistent with the typical treatment refractoriness of personality disorders (Abraham & Calabrese, 2008). Another limitation is accurately diagnosing and assessing the severity of MDD in ASD populations, where clinical presentation may be atypical and standardized measures may not be validated. To assess depression severity, we used clinician and self-report measures, including input from caregivers when available. This approach aligns with previous studies that suggest a multi-informant assessment in ASD captures complex symptoms and experiences more effectively (Sandercock et al., 2020).

## Conclusion

This exploratory study provides support for the safety and efficacy of ATBS in addressing treatment resistant MDD in autistic individuals. We observed rapid and large treatment effects across multiple domains that endured over time, with 56% (5/9) of subjects meeting criteria for remission at 12-weeks post-treatment. The distinctive treatment challenges posed by autistic populations, such as sensory hypersensitivities and the necessity of multi-informant outcome assessments, underscore the need for a nuanced approach to adapting ATBS protocols. Our findings suggest that neurocognitive testing could be an objective biomarker for predicting treatment response and potentially individualizing treatment. However, larger, sham-controlled studies are necessary to validate these findings. If successful, ATBS could emerge as an evidence-based intervention for MDD in ASD, an area that currently lacks effective treatments.

## Data Availability

The code is available at https://github.com/cincibrainlab.
